# Comprehensive Experimental Investigation of Rosehip (*Rosa canina* L.) Seed Oil as a Novel Biodiesel Feedstock: Engine Performance, Combustion, Emission, and Exergy Analyses

**DOI:** 10.1002/gch2.70143

**Published:** 2026-08-12

**Authors:** Erdoğan Güner

**Affiliations:** ^1^ Engineering Faculty Department of Mechanical Engineering Atatürk University Erzurum Türkiye

**Keywords:** biodiesel, bsfc, heat release, imep, NO_x_ emissions, *Rosa canina*

## Abstract

Given the increasing volatility of global energy markets, identifying previously unexplored resources like rosehip (*Rosa Canina* L.) is essential to diversify and strengthen the sustainable biodiesel feedstock portfolio. In this study, rosehip seed oil methyl ester (RSOME) was produced via transesterification at 50°C for 60 min using a 6:1 methanol‐to‐oil molar ratio and 1 wt.% KOH catalyst, achieving a biodiesel yield of 93.63%, and was experimentally evaluated in a four‐cylinder common‐rail CI engine over an engine speed range of 1500–3000 rpm under fixed‐load conditions. Combustion, performance, exergy, and emission characteristics were evaluated. RSOME exhibited a cetane number of 52.36. Among the tested fuels, B5 achieved the best overall performance, improving BSFC by 1.2%–5.5%, increasing exergetic efficiency by 1.6%–5.9%, and reducing exergy destruction by 1.3%–8.5% relative to diesel. B5 also exhibited speed‐dependent combustion behavior, slightly decreasing Pmax (up to 2.6%) at lower speeds but increasing it by 6.0% while reducing NO_x_ emissions by 6.8% at 3000 rpm. However, the large‐scale utilization of RSOME as a biodiesel feedstock may be constrained by the use of rosehip seed oil in high‐value health and cosmetic applications. Future studies should investigate RSOME under varying load conditions and long‐term engine durability scenarios.

## Graphical abstract

Rosehip seed oil is converted into biodiesel (Rosehip Seed Oil Methyl Ester‐RSOME) and experimentally evaluated in a diesel engine. Comprehensive analyses of engine performance, emissions, combustion characteristics, and exergy reveal the effects of RSOME blends on engine behavior, providing insights into the utilization of this novel biodiesel feedstock in compression ignition engines.

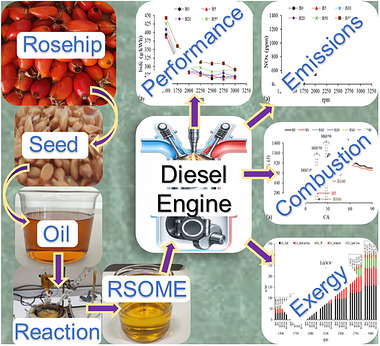

## 1 Introduction

Global energy demand continues to increase with population growth, urbanization, and expanding industrial activity. At present, a large share of the world's energy demand is still met by fossil fuels, particularly petroleum‐derived fuels in the transportation sector. However, reliance on fossil fuels is associated with key disadvantages, including energy crisis, exposure to price volatility, and environmental impacts. In compression ignition (CI) engines, the dominant use of fossil‐derived diesel contributes to both greenhouse gas emissions and urban air pollution, which has intensified research efforts toward renewable fuels that can be integrated into existing diesel engine infrastructure with minimal modification.

Biodiesel, composed mainly of fatty acid methyl esters (FAME), has emerged as one of the most feasible renewable alternatives because of its biodegradability, oxygenated molecular structure, and compatibility with CI engines. Because biodiesel can be produced from a wide variety of lipid feedstocks and utilized in existing CI engines with little or no hardware modification, it has attracted considerable attention as a practical renewable fuel. Compared with diesel fuel, biodiesel is oxygenated and sulfur‐free, typically has a higher flash point, and can improve fuel lubricity; consequently, biodiesel use is widely reported to reduce incomplete‐combustion‐related pollutants such as CO [1, 2], unburned hydrocarbons (UHC) [3, 4], and smoke opacity [5, 6], while its lower heating value (LHV) relative to diesel [7, 8] may increase brake specific fuel consumption (BSFC) in many cases [9, 10].

Feedstock selection plays a decisive role in determining the sustainability, production feasibility, and fuel properties of biodiesel. Accordingly, biodiesel feedstocks are commonly classified into generations, reflecting both fuel property differences and broader sustainability considerations (land use, food competition, and lifecycle emissions). Because sustainable scale‐up strongly depends on resource availability and land‐use constraints, biodiesel feedstocks are commonly categorized by “generations” [11]. First‐generation biodiesel is produced from edible vegetable oils, which can raise the food‐versus‐fuel concern [12, 13]. Second‐generation biodiesel relies on non‐edible oils, waste lipids (e.g., waste cooking oil), and other low‐cost feedstocks that can alleviate competition with food supply and potentially improve sustainability [14, 15]. Third‐generation biodiesel is typically associated with microalgae‐derived lipids, which offer high areal productivity but face challenges in cultivation, harvesting, and downstream processing [16, 17, 18]. Fourth‐generation concepts extend to more advanced routes, including genetically improved organisms and integrated systems aiming for higher yields and improved carbon management [19, 20].

Although numerous studies have explored biodiesel derived from common non‐edible feedstocks such as jatropha [21, 22], waste cooking oil [23, 24], and animal fats [25, 26], attention has recently turned toward underutilized seed oils that are by‐products of agricultural or food‐processing industries. In the face of growing concerns over both global energy security and food security, the identification and characterization of novel non‐edible feedstocks for biodiesel production have become increasingly important to ensure sustainable fuel production without intensifying the food‐versus‐fuel conflict. In this context, rosehip seed oil obtained from *Rosa Canina* L. represents a promising yet largely unexplored resource. Recent studies have also suggested that rosehip seed oil possesses suitable physicochemical characteristics for biodiesel production, highlighting its potential as a renewable feedstock [27]. Existing literature on rosehip seed oil has largely emphasized its fatty acid profile and antioxidant properties for cosmetic and nutraceutical applications, whereas its application as a biodiesel feedstock and its behavior in CI engine combustion systems have received very limited attention.

Despite these limitations, the recovery of discarded rosehip seeds from domestic and industrial processing, together with the utilization of surplus or residual rosehip seed oil generated by the health and cosmetic industries, could substantially improve its practical feasibility. Therefore, rosehip seed oil remains a promising non‐edible feedstock for sustainable biodiesel production.

Considering both its potential as a sustainable non‐edible feedstock and the limited knowledge regarding its engine application, the present study investigates RSOME as a second‐generation biodiesel candidate through an integrated assessment of four complementary aspects: (i) engine performance (BSFC and BTE), (ii) regulated emissions (CO, HC, NO_x_, and exhaust temperature), (iii) combustion characteristics and cyclic stability based on in‐cylinder pressure analysis (pressure trace, max(dP/dθ), cumulative net heat release, MBF10/50/90, and COV_IMEP_), and (iv) thermodynamic performance through second‐law (exergy) analysis.

Beyond conventional (first‐law) performance indicators, second‐law (exergy) analysis has gained increasing attention in CI engine studies because it enables the quantification of thermodynamic irreversibilities and the distribution of energy losses within the engine system. Consequently, exergy‐based metrics complement conventional performance indicators such as brake specific fuel consumption (BSFC) and brake thermal efficiency (BTE), providing a more comprehensive assessment of the thermodynamic performance and sustainability of alternative fuels.

To place the present study within the broader context of biodiesel research, Table 1 summarizes representative studies published since 2020 on biodiesels derived from various novel feedstocks, highlighting the combustion, performance, and emission parameters commonly investigated.

**TABLE 1:** Summary of recent studies on biodiesel fuel from novel feedstocks (2020 onward).

Refs.	Biodiesel type	Key results
[28]	Chia (*Salvia Hispanica* L.) seed oil methyl ester (CSME); Blends: CSME10, CSME20, CSME30	A conversion yield of 96.58% was reported. The CSME10 blend exhibited a brake thermal efficiency (BTE) of 30.29%, which was very close to that of diesel fuel. Compared to diesel, CSME10 led to reductions of 1.24% in NO_x_, 1.51% in HC, 3.03% in CO, and 2.14% in smoke.
[29]	Wild Oak (*Quercus brantii* Lindl) oil (QBLO); Blends: B5, B10, B15, B20	Yield: 95.75% (Co@CuO nanocatalyst). The B20 blend provided improved performance and emission characteristics. Increasing biodiesel concentration led to higher BSEC and EGT, accompanied by a decrease in BTE.
[30]	*Pyrus glabra* (Anchochek) seed oil; Blends: B5, B10, B15, B20	The optimum operating point corresponded to 70% engine load and 9% biodiesel content. At this point, BTE reached 20%, and BSFC was 307 g/kW.h, while emissions were measured as 12 ppm (HC) and 209 ppm (NO_x_).
[31]	Coconut fatty acid distillate (CFAD) biodiesel (CFAB); Blends: CFAB20, 40, 60, 100	A maximum yield of 92.6%. The performance of CFAB20 was the closest to diesel. At full load, CFAB100 reduced CO by 50%, HC by 36.6%, and smoke by 42.9%, though NO_x_ emissions increased due to improved combustion.
[32]	*Lilium ledebourii*, *Iris meda*, and *Viola odorata* seed methyl esters; Blends: B20, B50, B80, B100	B20 blends provided torque values very close to diesel fuel. *Iris meda* biodiesel exhibited the lowest BSFC. B100 usage resulted in a massive smoke opacity reduction of 69%–74%, while *Viola odorata* caused the highest NO_x_ increase (29.5%).
[33]	*Koelreuteria paniculata* (KP) seed oil biodiesel	KP seed oil content was reported to be 28%–30%, with a low free fatty acid (FFA) level of 0.91%. Optimized base‐catalyzed transesterification resulted in a 95.2% yield. The resulting fuel satisfied ASTM D6751 and EN 14214 specifications.
[34]	Cocklebur (Cocklebur sp.) seed oil biofuel	Seed oil content was determined as 37.2%. Using ZnO nanoparticles as a catalyst, a maximum conversion yield of 93.33% was achieved. The structural characterization and fuel properties were found to be fully compatible with international standards.
[35]	*Bauhinia racemose* biodiesel (BRB); Blends: PD10, 20, 30 and water emulsions (PD30BRB5W, 10 W)	A maximum yield of 92.9% was achieved under optimized input parameters. While adding 30% BRB alone decreased BTE by 3.2%, the 10% water‐emulsified blend improved BTE by 3.9% and reduced NO_x_ emissions by 12.1%.
[36]	Wild Olive Oil (*Olea ferruginea*) biodiesel (WOO)	Wild olive oil was evaluated as a novel non‐edible feedstock, with an oleic acid content of 57.42%. Physicochemical characterization according to ASTM and EN methods confirmed its suitability as a sustainable bioenergy resource.
[37]	*Terminalia chebula* methyl ester (TCME); Blends: B10, B20, B30, B40, B100	A yield of 97.1% was obtained using green‐synthesized CuO nanoparticles. The B20 blend showed the best performance results (BTE of 31.34%). At full load, B20 reduced CO by 35.3% and HC by 23.26%, but NO_x_ emissions rose by 23.17%.
[38]	Lal ambari (*Hibiscus sabdariffa*) seed oil biodiesel; Blends: LA20, LA40, LA60, LA80, LA100	At full load, the LA20 blend showed a 2.15%–2.5% decrease in BTE and a 4.4% increase in BSFC compared to diesel. Significantly reduced smoke (16.6%) and NO_x_ (1.52%) for the LA20 blend, while CO_2_ emissions increased by 3.5%. HRR and peak cylinder pressure were lower for all biodiesel blends compared to diesel.
This study	Rosehip Seed Oil Methyl Ester (RSOME); Blends: B5, B10, B20, B50, B100	RSOME demonstrated superior ignition quality owing to its high cetane number (52.36) and oxygen content (11.46%). The B5 blend achieved an improvement in BSFC ranging from 1.2% to 5.5% and in exergetic efficiency between 1.6% and 5.9%, while reducing exergy destruction by 1.3% to 8.5%. Although the B5 fuel reduced P_max_ by up to 2.6% at low speeds, it provided a 6% increase at 3000 rpm. Similarly, under the same condition, it reduced NO_x_ emissions by 6.8%.

Despite the growing number of studies on non‐edible biodiesel feedstocks, a clear knowledge gap remains regarding biodiesel derived from rosehip seed oil. In particular, although rosehip seed oil has been proposed as a potential biodiesel feedstock, its combustion behavior, thermodynamic performance, and regulated emissions in CI engines remain largely unexplored. Furthermore, no study has yet reported an integrated assessment combining in‐cylinder pressure‐based combustion diagnostics, engine performance, exergy efficiency, together with exergy distribution, and exhaust emission analysis.

To address this gap, the present study provides the first comprehensive experimental evaluation of rosehip seed oil methyl ester (RSOME) in a common‐rail CI engine using diesel (B0), RSOME‐diesel blends (B5, B10, B20, and B50), and neat RSOME (B100). By integrating combustion diagnostics, engine performance, exergy analysis, and regulated emission measurements within a single experimental framework, this study provides a comprehensive thermodynamic and combustion characterization of RSOME in CI engines.

Specifically, the objectives of this study are to: (i) produce and characterize RSOME; (ii) evaluate its effects on engine performance, combustion characteristics, cyclic combustion stability, and regulated exhaust emissions under different operating conditions; and (iii) perform an exergy analysis to quantify exergetic efficiency and exergy distribution.

## 2 Materials and Method

### 2.1 Feedstock and Biodiesel Production

**FIGURE 1:**
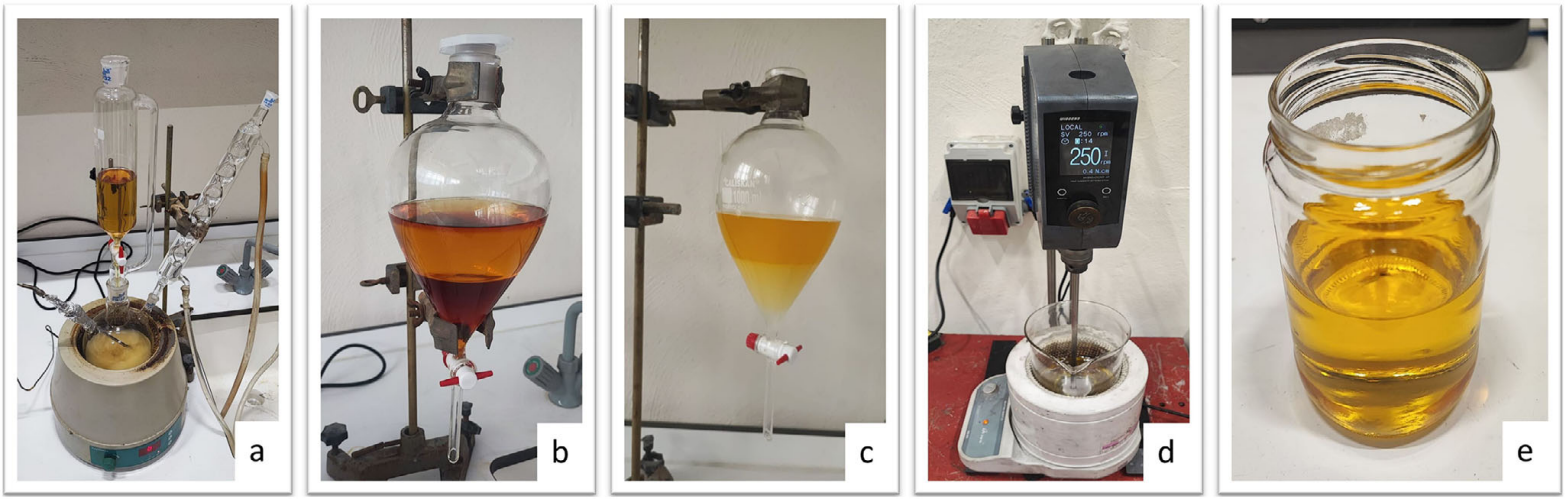
Photographs of the RSOME production process: (a) transesterification setup, (b) phase separation after reaction, (c) biodiesel (upper phase) and glycerol (lower phase) after settling, (d) washing/mixing stage, and (e) final RSOME product.

Rosehip seed oil was purchased from a commercial supplier and used as the feedstock for biodiesel production. Biodiesel (rosehip seed oil methyl ester, RSOME) was produced via base‐catalyzed transesterification using methanol as the alcohol and potassium hydroxide (KOH) as the catalyst (1% wt. of oil). The alcohol‐to‐oil molar ratio was set to 6:1. The reaction was conducted at 50°C for 60 min. The main steps of the biodiesel production process are illustrated in Figure 1.

KOH was selected as the alkaline catalyst in this study because homogeneous alkaline catalysts generally provide higher transesterification rates and biodiesel yields under relatively mild reaction conditions than acid‐catalyzed processes. Among alkaline catalysts, KOH exhibits high catalytic activity, good solubility in methanol, and enables efficient conversion of triglycerides into fatty acid methyl esters with comparatively short reaction times.

Under these conditions, three independent production runs gave an average biodiesel yield of 93.63%, with a standard deviation of 0.19%. The individual yields were 93.76%, 93.71%, and 93.42%; the yield ranged from 93.42% to 93.76%, corresponding to a maximum repeat‐to‐repeat difference of 0.34% points.

### 2.2 Fuel Property Measurements

The physicochemical properties of the produced RSOME and petroleum diesel are summarized in Table 2, alongside the respective international standard limits (EN 14214). The fuel parameters, including density, kinematic viscosity, cold filter plugging point (CFPP), and lower heating value (LHV), were experimentally determined at the Fuel Analysis Laboratory, Department of Chemical Engineering (Atatürk University, Erzurum, Turkey) in accordance with the relevant standard test methods. Conversely, the cetane number (CN), iodine value (IV), and oxygen content (O) were theoretically calculated with Equations (1), (2), and (3) based on the fatty acid methyl ester (FAME) composition detailed in Table 3. The physicochemical properties of the reference ultralow sulfur diesel fuel (B0) utilized in this study were strictly governed by the EN 590 standards, which define the technical requirements and testing protocols for automotive diesel fuels.

**TABLE 2:** Fuel properties of RSOME and petroleum diesel in comparison with international standards.

Parameter	Unit	Diesel	RSOME	Standard/Method	Biodiesel Std. (EN 14214)	Diesel Std. (EN 590)
Density (15°C)	kg m^−3^	832	883	EN ISO 12185	860–900	820–845
Kinematic viscosity (40°C)	mm^2^ s^−1^	2.75	4.163	EN ISO 3104	3.5–5.0	2.0–4.5
Cold filter plugging point (CFPP)	°C	−5	−11	EN 116	variable	variable
Lower heating value (LHV)	MJ kg^−1^	42.8	39.08	EN ISO 18125	—	—
Cetane Number (CN)	—	51.0	52.36	GC	min. 51.0	min. 51.0
Iodine Value (IV)	g I_2_/100g	—	135.5	GC	max. 120	—
Oxygen Content (O)	wt.%	0	11.46	GC	—	—

**TABLE 3:** Fatty acid methyl ester (FAME) composition and individual cetane numbers of the produced RSOME.

Acid	Area (%)	CN (‐)	Refs.
Caprylic (C8:0)	0.0217	33.6	[39]
Myristic (C14:0)	0.1106	72.0	[40]
Palmitic (C16:0)	6.1142	91.0	[40]
Palmitoleic (C16:1)	0.0512	51.0	[41]
Stearic (C18:0)	1.9691	101.0	[41]
Oleic (C18:1)	33.9301	59.3	[41]
Linoleic (C18:2)	56.9422	42.2	[42]
Alfa‐Linolenic (C18:3)	0.0665	45.9	[43]
Arachidic (C20:0)	0.2795	112.0	[44]
Eicosenoic (C20:1)	0.163	73.2	[44]
Eicosapentaenoic (C20:5)	0.3519	22.0	[44]
**Total**	**100**		

The cetane number and iodine value were determined from the FAME profile using Equation (1) [45] and Equation (2) [46], respectively, resulting in a calculated cetane number of 52.36 and iodine value of 135.5 for RSOME. The elemental oxygen content (O) of the RSOME was calculated theoretically using Equation (3) based on the molecular weight of each FAME component:

(1) $$CN_{mix}=\sum A_{c}\times CN_{C}\;$$

where *CN_mix_* and *CN_C_* is the cetane number of the mixture and the individual ester, respectively, *A_c_* refers to the relative fraction (%) of each ester in the mixture.

(2) $$IV=\sum \frac{254DA_{i}}{MW_{i}}\;$$

where *D* is the number of double bonds, *A_i_* is the percentage (%) of the component, and *MW_i_* is the molecular weight of the corresponding component.

(3) $$O=\sum \frac{32}{MW_{i}}W_{i}\;$$

where *MW_i_* is the molecular weight and *W_i_* is the mass fraction of each individual fatty acid methyl ester.

To facilitate a direct comparison between the original feedstock and the produced biodiesel, the key fuel properties of rosehip seed oil (RSO) and rosehip seed oil methyl ester (RSOME) are summarized in Table 4.

**TABLE 4:** Comparison of the key fuel properties of rosehip seed oil (RSO) and rosehip seed oil methyl ester (RSOME).

Parameter	Unit	RSO	RSOME	Standard/Method
Density (15°C)	kg m^−3^	921.6	883	EN ISO 12185
Kinematic viscosity (40°C)	mm^2^ s^−1^	33.637	4.163	EN ISO 3104

As shown in Table 4, the kinematic viscosity of rosehip seed oil (RSO) decreased from 33.637 to 4.163 mm^2^ s^−^^1^ after transesterification, representing an approximately eightfold reduction. This pronounced decrease confirms the effectiveness of the transesterification process, whose primary objective is to convert highly viscous vegetable oils into fatty acid methyl esters with fuel properties suitable for compression ignition engines.

### 2.3 Engine Test Setup and Procedure

**FIGURE 2:**
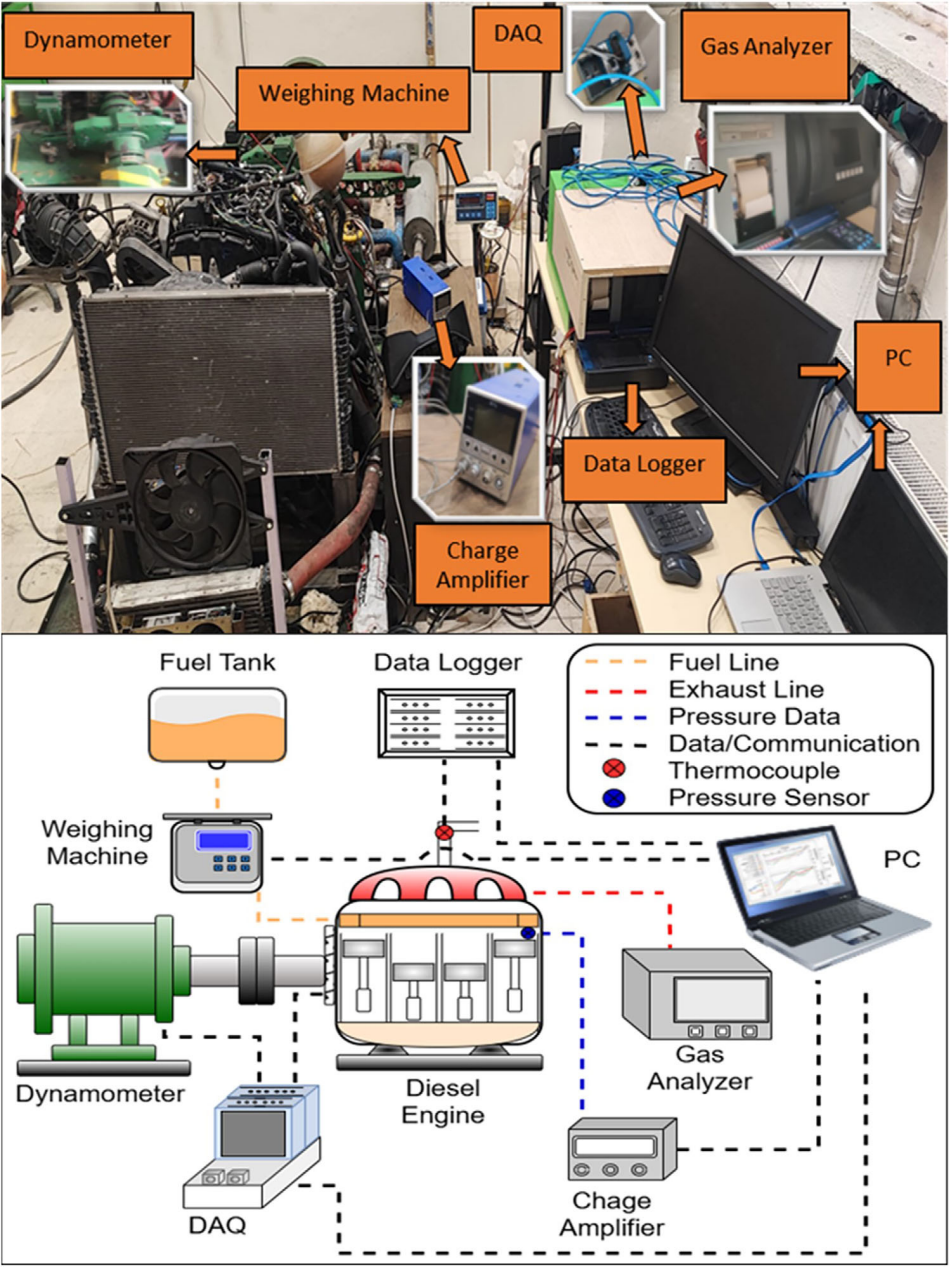
Photograph and schematic representation of the experimental engine test setup.

Engine experiments were performed using diesel (B0), RSOME‐diesel blends (B5, B10, B20, and B50), and neat RSOME (B100) under a constant dynamometer load setting (%25). The engine speed was varied from 1500 to 3000 rpm in 250 rpm increments (1500, 1750, 2000, 2250, 2500, and 3000 rpm). Each operating condition was tested in duplicate. The engine test setup used in this study is shown in Figure 2, while the specifications of the test engine and the measurement instruments used throughout the experimental study are summarized in Tables 5 and 6, respectively.

**TABLE 5:** Test engine specifications.

Parameter	Value
Cylinders	4‐cylinder, inline
Maximum power	125 PS (92 kW) (@3500 rpm)
Maximum torque	330 Nm
Stroke	94.6 mm
Bore	86 mm
Displacement	2.198 L
Compression ratio	15.5:1
Fuel injection system	Common‐rail direct injection
Injector type	Piezo injector

**TABLE 6:** Specifications of the measurement instruments used in the experimental setup.

Instrument	Manufacturer	Model	Parameter	Key Specification
Gas Analyzer	Bosch	BEA 250	HC, CO	Resolution: 1 ppm (HC), 0.001% (CO)
NO_x_ Sensor Module Kit	NI	9755	NO_x_	Accuracy: 10 ppm (< 100 ppm), ±10% (> 100 ppm)
Weighing Machine	TEM	BT1TA	Fuel mass	e = 0.5 g
Pressure Transducer	Kistler	6052C	In‐cylinder Pressure	≈ −20 pC/bar
Crank Angle Encoder	AVL	365C	Crank Angle	Static assignment accuracy: 0.1 deg CA
Charge Amplifier	AVL	FlexIFEM	Pressure signal	—
Data Logger	Hioki	LR8401‐20	Temperature signal	—
DAQ	NI	cRio 9063	Data acquisition	—

### 2.4 Energy and Exergy Analysis

Energy and exergy analyses were conducted for the control volume defined by the test engine. Performance metrics were evaluated using the following definitions:

(4) $$BSFC=\frac{{\dot{m}}_{f}}{P_{b}}\;$$

(5) $$BTE=\frac{P_{b}}{{\dot{m}}_{f}.H_{u}}\;$$

where ${\dot{m}}_{f}$ is fuel mass flow rate, H_u_ is lower heating value and P_b_ is brake power.

The steady‐state rate balances of mass and energy are written as [47]:

(6) $$\sum {\dot{m}}_{in}-\sum \;{\dot{m}}_{out}=0$$

(7) $$\dot{Q}+\dot{W}=\sum {\dot{m}}_{out}h_{out}-\sum {\dot{m}}_{in}h_{in}$$

where $\dot{Q}$ and $\dot{W}$ are the heat and work rates, respectively.

Brake power is expressed as:

(8) $$\dot{W}=\omega .T=\frac{2\pi NT}{60}\;$$

where ω is angular velocity, T is torque, and N is engine speed (rpm).

The rate of fuel energy input is:

(9) $${\dot{E}}_{f}={\dot{m}}_{f}\;.H_{u}$$

Accordingly, the (first‐law) brake thermal efficiency is:

(10) $$\eta_{t}=\frac{\dot{W}}{{\dot{E}}_{f}}\;$$

The exergy rate balance for the control volume can be written as:

(11) $$\dot{E}x_{heat\_loss}+\dot{E}x_{W}=\sum \dot{E}x_{in}-\sum \dot{E}x_{out}-\dot{E}x_{dest}$$

where $\dot{E}x_{\mathrm{dest}}$ is the exergy destruction rate. The exergy work rate is taken as the brake power, i.e., $\dot{E}x_{W}$ = $\dot{W}\;$. The total exergy rate entering the control volume is expressed as:

(12) $$\sum \dot{E}x_{in}=\dot{E}x_{f}+\dot{E}x_{a}$$

where $\dot{E}x_{f}$ and $\dot{E}x_{a}$ are the fuel and air exergy rates, respectively.

Fuel exergy is evaluated as:

(13) $$\dot{E}x_{f}={\dot{m}}_{f}\;.e_{f}$$

(14) $$e_{f}=H_{u}\;.\varphi$$

where *e_f_* is the specific chemical exergy of the liquid fuel, and φ is the chemical exergy factor per unit mass [48]:

(15) $$\varphi =1.0401+0.1728\frac{h}{c}+0.0432\frac{o}{c}+0.2169\frac{\alpha }{c}\left(1-2.0628\frac{h}{c}\right)$$

In the present analysis, the thermomechanical and chemical exergy of intake air were assumed to be negligible relative to the fuel chemical exergy.

The exergy leaving the control volume is taken as the exhaust exergy:

(16) $$\sum \dot{E}x_{out}=\dot{E}x_{exh}\;$$

The exhaust exergy is computed as the sum of thermomechanical and chemical contributions:

(17) $$\dot{E}x_{exh}=\sum {\dot{m}}_{i}{\left(e_{th}+e_{ch}\right)}_{i}$$

where i denotes the exhaust‐gas species. The specific thermomechanical [47] and chemical exergies [49] are calculated as:

(18) $$e_{th}=\left(h-h_{0}\right)-T_{0}\left(s-s_{0}\right)$$

(19) $$e_{ch}=\bar{R}\;T_{0}\ln\left(\frac{y}{y_{0}}\right)$$

where T_0_ is the dead‐state temperature, (h_0_, s_0_) are the dead‐state properties, *y* is the molar fraction in the mixture, and *y*_0_ is the molar fraction in the environment.

Exergy associated with heat loss is evaluated by:

(20) $$\dot{E}x_{heat\_loss}=\sum \left(1-\frac{T_{0}}{T_{w,ave}}\right)\dot{Q}$$

(21) $$T_{w,ave}=\frac{\left(T_{cw,in}+T_{cw,out}\right)}{2}\;$$

where T_cw, in_ and T_cw, out_ are the inlet and outlet cooling‐water temperatures.

Finally, the exergetic efficiency and entrophy generation rate [50] are calculated as follows:

(22) $$\varepsilon =\frac{\dot{E}x_{W}}{\dot{E}x_{f}}\;$$

(23) $$\dot{S}=\frac{\dot{E}x_{dest}}{T_{0}}\;$$

### 2.5 Uncertainty Analysis

Measurement uncertainty was quantified using the standard propagation‐of‐uncertainty approach [51]. The assigned instrument uncertainties were: load ±0.1 kg, engine speed ±1 rpm, fuel‐mass measurement from the balance ±0.5 g (used to compute fuel consumption over a fixed sampling interval), and NO_x_ sensor uncertainty 10 ppm for NO_x_ < 100 ppm, ±10% for above 100 ppm.

For a derived variable R  =  R(x_1_, x_2_, . . ., x_k_), the combined standard uncertainty was calculated as:

(24) $$u_{R}=\sqrt{\sum_{k=1}^{n}{\left(\frac{\partial R}{\partial x_{k}}u_{x_{k}}\right)}^{2}}\;$$

All derived quantities (including P_b_, BSFC, and BTE) were evaluated by substituting the corresponding measured variables into Equation (24), without presenting intermediate algebraic expansions. NO_x_ uncertainty was taken directly from the dedicated sensor specification.

Accordingly, the final combined uncertainties obtained overall operating points are: $U_{P_{b}}\;$= (0.47–1.73)%, *U_BSFC_* = ±(0.56–2.44)%, *U_BTE_* = ±(0.56–2.44)%, and $U_{NO_{x}}$ = ±10% (> 100 ppm).

The overall experimental uncertainty was calculated by combining the maximum combined uncertainties of P_b_, BSFC, BTE, and NO_x_ using the root‐sum‐square method, as expressed in Equation (25), and was determined to be ±10.72%. This value is primarily governed by the manufacturer‐specified uncertainty of the NO_x_ sensor (±10% for concentrations above 100 ppm), which constitutes the dominant contribution to the overall experimental uncertainty.

(25) $$U_{overall}=\sqrt{{\left(U_{P_{b}}\right)}^{2}+{\left(U_{BSFC}\right)}^{2}+{\left(U_{BTE}\right)}^{2}+{\left(U_{NO_{x}}\right)}^{2}}\;$$

## 3 Results and Discussion

In this study, diesel‐engine performance, emission, and combustion characteristics were experimentally investigated for RSOME biodiesel over a range of engine speeds using different blend ratios, and a complementary exergy analysis was performed to quantify the associated second‐law efficiency trends and irreversibilities.

### 3.1 Performance Analysis

**FIGURE 3:**
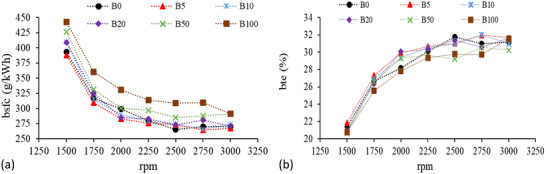
Comparison of BSFC and BTE for B0‐B100 fuels across the tested engine speed range.

Fuel performance was evaluated using brake specific fuel consumption (BSFC) and brake thermal efficiency (BTE), which are widely adopted indicators for quantifying, respectively, the fuel‐mass requirement per unit brake work and the effectiveness of converting the fuel's chemical energy into useful brake power. In the present analysis, BSFC trends are used to interpret the combined influence of blend heating value and operating conditions on the overall fuel demand, while BTE provides a normalized measure of conversion efficiency that enables a consistent comparison among diesel, RSOME‐diesel blends, and neat RSOME across the considered speed range. Figure 3 compares BSFC and BTE responses of the fuels over the investigated engine‐speed range.

As shown in Figure 3a, BSFC varies significantly with engine speed and blend ratio. Across all tested fuels, BSFC exhibits a sharp decline as the engine speed increases from 1500 to 2500 rpm, followed by a stabilization or slight increase at higher speeds. The initial reduction is primarily due to the improvement in volumetric efficiency and mechanical efficiency as the engine moves toward its optimal operating range. In general, increasing the RSOME fraction leads to a systematic increase in BSFC relative to B0. This trend is consistent with the lower heating value (LHV) of biodiesel; since biodiesel contains less energy per unit mass, a higher fuel mass flow is required to deliver the same brake power. Furthermore, the higher density and viscosity of RSOME blends affect the fuel injection pump's volumetric discharge, contributing to the higher mass‐based fuel consumption observed for B50 and B100.

Figure 3b presents the corresponding BTE trends, indicating that the conversion efficiency is sensitive to both speed and blend ratio. The BTE curves exhibit a speed‐dependent optimum, typically peaking around 2500 rpm. This behavior reflects the interplay between combustion duration, heat‐transfer losses, and friction/pumping losses as speed changes. At lower speeds (1500–1750 rpm), higher heat transfer losses to the cylinder walls reduce BTE, whereas at high speeds (3000 rpm), increasing mechanical friction and shorter residence times for complete combustion limit further efficiency gains. Differences among blends suggest that RSOME can shift the effective operating point of highest efficiency. While the fuel‐bound oxygen in RSOME can promote more complete combustion (potentially improving BTE), this chemical advantage is often counterbalanced by its inferior atomization characteristics due to higher viscosity. Consequently, higher blends like B100 show slightly lower BTE compared to B0 at most operating points, reflecting the dominance of physical constraints over chemical oxygenation benefits in terms of thermal energy conversion.

Considered together, Figure 3a,b demonstrates the expected inverse relationship between BSFC and BTE, as can also be inferred from Equations (4) and (5). Conditions that yield higher BTE tend to coincide with lower BSFC, as the more effective conversion of chemical energy into brake work reduces the required fuel mass. The observed BSFC penalty for higher RSOME fractions, even at points where BTE remains comparable, underscores the primary role of fuel energy density in governing mass‐based fuel economy.

The performance trends observed here are in high agreement with the overall emission characteristics discussed in the following section. Specifically, the efficiency limitations noted at certain operating points‐driven by the physical constraints of RSOME blends‐directly influence the resulting combustion quality and emission profiles across the tested speed range.

### 3.2 Emission Analysis

**FIGURE 4:**
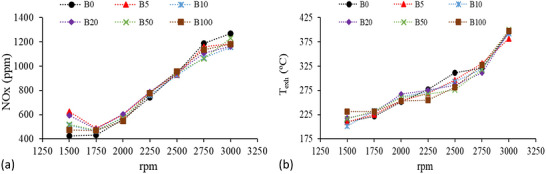
Impact of engine speed on NO_x_ emissions and exhaust gas temperature for various RSOME‐diesel blends.

Regulated emission characteristics were evaluated primarily in terms of NO_x_, HC, and CO to elucidate how RSOME blending and engine speed influence the competing pathways of thermal NO_x_ formation and incomplete‐combustion products under the investigated operating conditions. Figure 4 presents the coupled variation of NO_x_ emissions and exhaust gas temperature with engine speed for B0‐B100 fuels.

As shown in Figure 4a, NO_x_ emissions exhibit a pronounced dependence on both engine speed and blend ratio. A notable characteristic is the slight reduction in NO_x_ levels as the speed increases from 1500 to 1750 rpm, which can be attributed to enhanced in‐cylinder turbulence at this specific operating point. Improved mixing likely reduces the prevalence of localized high‐temperature “hot spots” where NO_x_ formation is most intense. However, beyond 1750 rpm, a sharp and consistent increase in NO_x_ is observed across all blends. This trend is initially driven by the thermal NO_x_ mechanism (Zeldovich mechanism), as the higher combustion frequency and energy throughput at elevated speeds significantly raise the peak cycle temperatures. Notably, as the speed exceeds 2250 rpm, this increase becomes even more pronounced. As further evidenced by the MBF phasing and burn duration analysis in Section 3.3, this intensified NO_x_ formation is closely linked to the advanced combustion onset (MBF10) and the extended residence time of gases at peak temperatures in the higher speed regimes.

Besides engine speed, the influence of RSOME blending ratio on NOx formation also depends on the prevailing combustion conditions. With increasing RSOME content, NO_x_ emissions do not exhibit a uniform trend over the entire operating range. At lower engine speeds, the oxygenated nature of RSOME may promote NO_x_ formation by enhancing local combustion temperatures. However, at higher engine speeds (2750–3000 rpm), lower NO_x_ emissions were observed for all RSOME blends. This behavior can be attributed to the combined effects of the high cetane number of RSOME (52.36), which shortens the ignition delay and suppresses excessive premixed combustion, together with its lower heating value, which may moderate the peak combustion temperature. Consequently, despite the oxygenated nature of the fuel, thermal NO_x_ formation governed by the Zeldovich mechanism is reduced under high‐speed operating conditions.

As illustrated in Figure 4b, the exhaust gas temperature T_exh_ increases systematically with engine speed, providing an integral indicator of the overall thermal state of the engine. The strong correlation between the rising trends of T_exh_ and NO_x_ after 1750 rpm confirms that NO_x_ formation in RSOME‐diesel blends is predominantly thermally governed. While biodiesel has a lower heating value (LHV) than B0, the slight variations in T_exh_ reflect a complex balance: the fuel's oxygen content enhances combustion efficiency and local temperatures, while its physical properties (viscosity and density) influence the timing of the heat release.

Considered together, Figure 4a,b offers a physically consistent interpretation of NO_x_ formation: operating points associated with elevated T_exh_ generally imply higher in‐cylinder temperatures and can therefore coincide with higher NO_x_ yield, whereas conditions that reduce the overall thermal level or shorten effective high‐temperature residence can suppress NO_x_ formation. Hence, the combined trends support the view that the NO_x_ behavior of RSOME blends is a result of the interplay between oxygen‐enhanced chemical kinetics and the engine's global thermal history, modulated by blend‐dependent combustion phasing.

After discussing thermally driven NO_x_ behavior, the analysis turns to incomplete‐combustion products to complete the emissions perspective.

**FIGURE 5:**
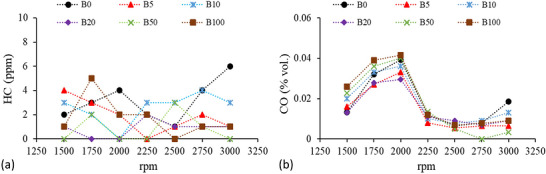
HC and CO emission profiles for B0 and RSOME blends under different engine speed conditions.

Figure 5 summarizes how HC and CO emissions evolve with speed as the RSOME blending ratio increases.

Figure 5a illustrates that HC emissions exhibit a non‐monotonic response to both engine speed and blend ratio, reflecting the complex interplay between fuel‐air mixing quality, local equivalence‐ratio distribution, and late‐cycle oxidation. While the oxygen content in biodiesel generally promotes HC oxidation in fuel‐rich or quench‐prone regions, the observed fluctuations suggest that the magnitude of this effect is highly dependent on the engine operating conditions. Factors such as altered ignition delay, spray penetration dynamics, and potential wall‐wetting sensitivity at specific engine speeds can offset the chemical advantages of oxygenated blends. Consequently, no single mechanism dominates HC formation over the entire operating range, leading to the partial overlap observed among the tested fuels. Furthermore, because the measured HC emissions remain at relatively low levels for all fuels, even small absolute variations become more apparent in the graphical representation, contributing to the observed scatter in the HC trends.

Figure 5b indicates that CO emissions are highly sensitive to both engine speed and blend ratio, reflecting the underlying competition between CO formation in fuel‐rich zones and its subsequent oxidation to CO_2_. For RSOME blends, although the fuel‐bound oxygen typically promotes oxidation, a notable increase in CO emissions is observed at lower speeds (1500–2000 rpm) compared to B0. This suggests that the chemical advantages of biodiesel (i.e., its oxygen content) are offset by its physical disadvantages, such as higher viscosity and density, which deteriorate atomization quality and lead to localized fuel‐rich pockets. At these specific speeds, the combination of larger droplet sizes and limited residence time for oxidation likely limits the completeness of the combustion process, preventing the full utilization of the extra oxygen provided by the biodiesel molecular structure.

To gain a deeper underlying understanding of how these performance and emission trends arise, the underlying in‐cylinder combustion characteristics are examined in the following section.

### 3.3 Combustion Analysis

Combustion behavior was quantified using a set of complementary in‐cylinder pressure‐based metrics that collectively characterize load‐carrying capability, pressure‐rise severity, burn phasing, and the evolution of heat release. Specifically, IMEP was employed as the primary indicated‐work parameter to reflect cycle‐averaged combustion effectiveness, while P_max_ provides a direct indicator of peak firing pressure and the associated mechanical/thermal loading of the engine. The maximum pressure rise rate, max(dP/dθ), was used to assess the rapidity of energy release and the propensity for harsh combustion (combustion “sharpness”), which is closely linked to ignition delay, premixed‐burn fraction, and perceived combustion noise. In addition, mass fraction burned (MBF) phasing parameters were used to describe the progression of combustion in crank‐angle terms (e.g., early‐, mid‐, and late‐burn locations), thereby enabling a consistent comparison of burn timing and duration among diesel and RSOME blends. Finally, crank‐angle‐resolved cumulative net heat release analysis (CNHR) was adopted to interpret how the rate and distribution of chemical energy conversion shift with blend ratio and engine speed, providing mechanistic insight beyond the integral performance metrics.

**FIGURE 6:**
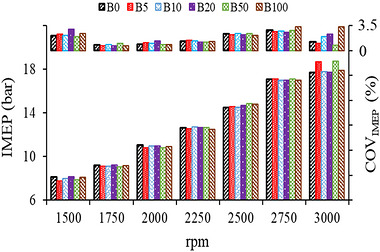
IMEP and cycle‐to‐cycle combustion stability (COV_IMEP_) for all fuel types across the tested conditions.

To ensure statistical reliability and capture the cyclic nature of the combustion process, in‐cylinder pressure data were recorded and averaged over 1000 consecutive cycles for each fuel and engine speed combination. To evaluate combustion stability, the coefficient of variation of IMEP (COV_IMEP_) was used as a standard cyclic‐variability indicator. Because COV_IMEP_ captures cycle‐to‐cycle fluctuations in indicated work, it provides a compact measure of repeatability and misfire/partial‐burn tendency, and it enables an objective assessment of whether RSOME blending materially alters the steadiness of combustion across the investigated operating range. To evaluate combustion repeatability, Figure 6 reports COV_IMEP_ for diesel, neat RSOME, and all blends under the tested conditions.

Figure 6 summarizes the coefficient of variation of the indicated mean effective pressure (COV_IMEP_), which is a widely used measure of cycle‐to‐cycle combustion stability in compression ignition engines. Low COV_IMEP_ values indicate repeatable indicated work output and, consequently, a stable combustion process with reduced propensity for partial‐burn events. Notably, the COV_IMEP_ values remained below 3.5% across all operating conditions, which is well within the acceptable limit for stable combustion in CI engines. These results show that, within the investigated operating range, the RSOME blends do not introduce a pronounced deterioration in combustion stability relative to the diesel baseline (B0). This indicates that despite the higher viscosity of RSOME, the fuel injection system maintains high cyclic repeatability, ensuring operational smoothness without compromising the engine's NVH (noise, vibration, and harshness) behavior or the robustness of emission‐control performance. Therefore, the observed stability supports the practical feasibility of RSOME blending from an engine‐operability perspective.

**FIGURE 7:**
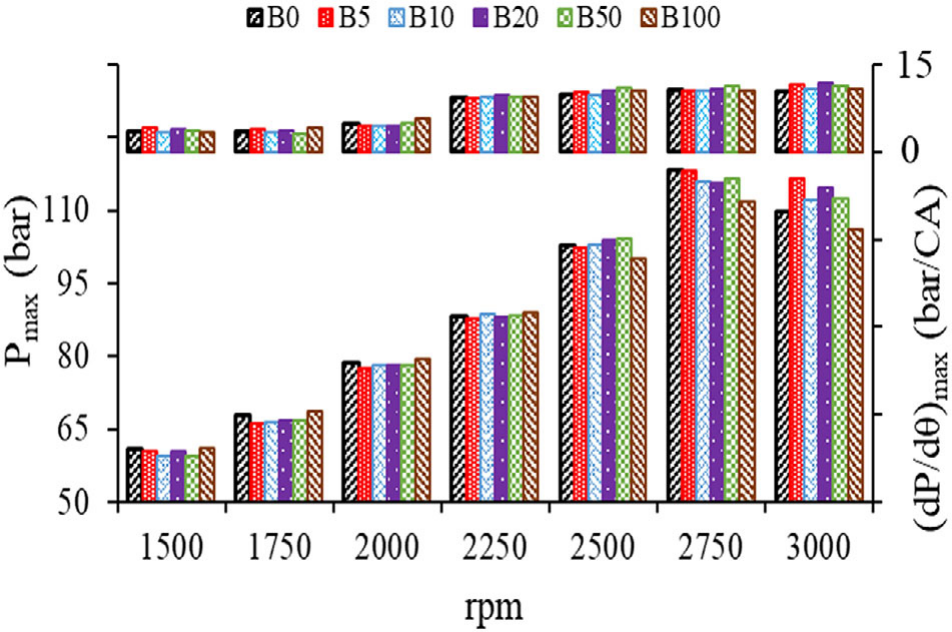
Peak in‐cylinder pressure (P_max_) and maximum pressure rise rate (max(dP/dθ)) for B0‐B100 fuels under the investigated operating conditions.

To complement the cycle‐variability assessment, the pressure‐domain characteristics of combustion are examined next. Figure 7 provides the in‐cylinder pressure traces together with pressure‐rise characteristics for B0 and RSOME‐fueled operation.

Figure 7 presents the in‐cylinder pressure response together with the corresponding pressure‐rise behavior, which are key indicators of combustion phasing and the intensity of the premixed burn fraction. The peak pressure level reflects the magnitude and timing of the main heat‐release event, whereas a higher maximum pressure‐rise rate is generally associated with more abrupt energy release that can increase combustion noise and mechanical loading. Relative to B0, RSOME blends can modify these characteristics because biodiesel alters spray formation and evaporation (via changes in viscosity and volatility) and can influence ignition delay through its different chemical reactivity and fuel‐borne oxygen.

Consistent with these fundamental drivers, as observed in Figure 7, while P_max_ reaches its peak at 2750 rpm, a slight reduction is noted at 3000 rpm across most blends. This is likely due to the increased friction losses and shorter time available for combustion at the highest engine speed. Interestingly, the maximum pressure rise rate max(dP/dθ), remains relatively stable between 2500 and 3000 rpm, suggesting that the acceleration of energy release reaches a plateau, where the chemical promotion of biodiesel (oxygen content) is balanced by the physical constraints of high‐speed operation. These observations are important because they connect the measured performance and emission trends to underlying in‐cylinder thermodynamic behavior and provide a practical basis for assessing operability constraints (e.g., acceptable pressure‐rise levels) as the RSOME fraction is increased.

**FIGURE 8:**
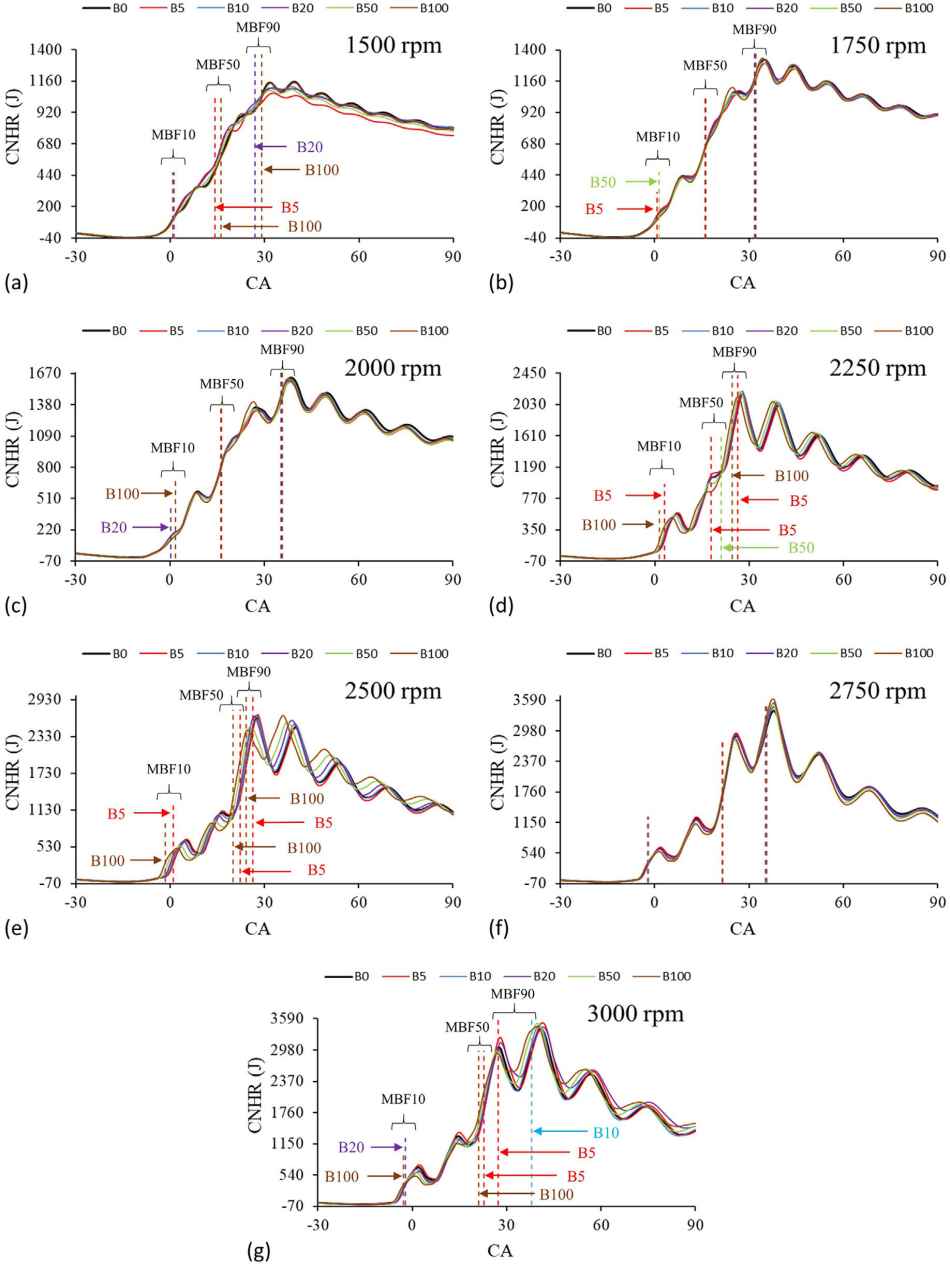
Crank‐angle‐resolved cumulative net heat release (CNHR) as a function of engine speed for all fuel types.

Following the pressure‐based interpretation, heat‐release phasing is examined to resolve how the combustion event is temporally distributed. Combustion‐phasing differences are further clarified in Figure 8 through cumulative net heat‐release (CNHR) profiles at different speeds. Additionally, the fuels that reach MBF10, MBF50, and MBF90 the earliest and the latest are indicated on the CNHR plots. As seen in Figure 8a, the fuels reaching MBF10 at the earliest and latest timings are nearly superimposed, i.e., they attain this condition at almost the same crank angle (CA). Therefore, to avoid visual clutter, fuel cases corresponding to MBF10, MBF50, and MBF90 that overlap (or are nearly identical) are not annotated separately on the plots; only conditions with clearly distinguishable differences are highlighted.

As a notable technical detail, the CNHR profiles in Figure 8 show distinct pressure‐induced oscillations following the main heat release peak, particularly at mid‐to‐high speeds. These oscillations reflect the high energy throughput and intensified turbulence within the combustion chamber.

Figure 8 presents the crank‐angle‐resolved cumulative net heat release (CNHR) histories and highlights how RSOME blending modifies the temporal distribution of chemical energy conversion. In general, the location of the main CNHR peak reflects the phasing of the dominant heat‐release event, whereas the peak magnitude is indicative of the intensity of the premixed burning fraction. The nearly superimposed MBF10 points across different blends further underscore that RSOME maintains a consistent start of combustion, which is vital for engine control stability. Moreover, the post‐peak tail of the CNHR curve characterizes the duration of diffusion‐controlled combustion and the completeness of late‐cycle oxidation.

The observed blend‐dependent shifts in CNHR can be attributed to biodiesel‐related changes in spray atomization and evaporation (via viscosity and volatility effects), as well as differences in chemical reactivity that influence ignition delay and the partitioning between premixed and mixing‐controlled burning. From an engine‐operability perspective, these CNHR trends provide mechanistic support for the performance and emission results reported in this study, because heat‐release phasing and rate directly govern in‐cylinder temperature history (and thus NO_x_ formation) while also affecting the oxidation potential for CO and HC. Overall, Figure 8 indicates that RSOME blends can alter combustion development without introducing abnormal heat‐release behavior within the investigated operating window.

Similar characteristics are also observed in the heat‐release results reported by Gumus [52], Hoang et al. [53], and Özçelik et al. [54], where the curves corresponding to different fuel types or blending ratios remain closely spaced and exhibit similar overall trends. Furthermore, the oscillation pattern and amplitude of the heat‐release curves vary with engine operating conditions, consistent with the behavior observed in Figure 8 of the present study.

**FIGURE 9:**
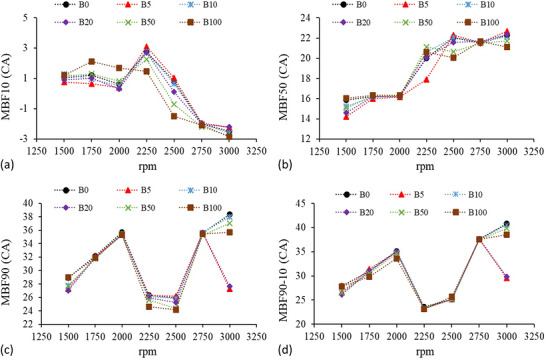
Mass fraction burned (MBF) phasing metrics (MBF10, MBF50, MBF90) and burn duration (MBF90‐10) for B0‐B100 fuels at different engine speeds.

Figure 9 compiles MBF‐based phasing indicators (MBF10, MBF50, MBF90) and burn duration (MBF90‐MBF10) across operating speeds.

Figure 9 summarizes the mass fraction burned (MBF) phasing metrics and the corresponding burn duration, thereby providing a compact and quantitative description of combustion timing and duration. MBF10 is commonly interpreted as an indicator of early flame development/combustion onset, whereas MBF50 represents the mid‐burn location and is frequently used as a primary combustion‐phasing metric linked to efficiency and NO_x_ formation via the in‐cylinder temperature history. MBF90 reflects late‐burn completion, and the interval MBF90‐10 characterizes the overall burn duration.

As illustrated in Figure 9a,b, a diverging trend is observed beyond 2250 rpm; while MBF10 advances (decreases toward TDC) due to the high cetane number and presence of molecular oxygen in RSOME, the MBF50 phasing actually retards. The shortened ignition delay provided by the superior ignition quality ensures an earlier start of combustion; however, the subsequent main combustion event is slightly prolonged at higher speeds. Furthermore, Figure 9d underscores the mechanistical reason behind the previously discussed emission trends. Between 2250 and 2750 rpm, the burn duration (MBF90‐10) increases significantly for RSOME blends. This prolonged combustion, initiated earlier by the high cetane number but finishing later, subjects the cylinder gases to high temperatures for an extended residence time, which is the primary driver for the peak NO_x_ formation observed at these speeds.

However, at the maximum speed of 3000 rpm, RSOME blends‐particularly B100‐exhibit a shorter burn duration compared to B0. This suggests that the fuel‐bound oxygen effectively accelerates the late‐stage oxidation process even under time‐constrained high‐speed conditions. The results indicate that RSOME blending can shift these phasing locations and modify burn duration depending on engine speed, consistent with the CNHR trends and with biodiesel‐induced changes in spray formation, evaporation, and ignition delay. From a practical perspective, these MBF metrics provide an objective basis for assessing whether RSOME blends alter combustion duration in a manner that influences thermal efficiency, combustion noise, and the oxidation pathways governing CO and HC emissions.

Previous studies by Adib et al. [55], Pradeep et al. [56], and Selvam et al. [57] have likewise demonstrated that combustion‐related parameters exhibit different trends with variations in biodiesel blending ratio and engine operating condition owing to the combined influence of fuel physicochemical properties and in‐cylinder combustion characteristics.

With the combustion‐phasing and stability behavior established, the discussion is extended to a second‐law viewpoint through exergy analysis.

### 3.4 Exergy Analysis

**FIGURE 10:**
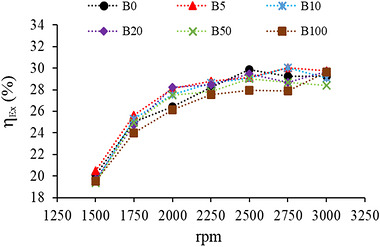
Exergetic efficiency for B0‐B100 fuels across the tested engine speed range.

Based on the exergy‐calculation equations given in Section 2.4, the exergetic efficiency (η_Ex_) is presented in Figure 10. This second‐law indicator complements the first‐law metrics (BSFC/BTE) by quantifying how effectively fuel exergy is converted into useful work. As observed in Figure 10, the η_Ex_ trends are strongly operating‐point dependent and closely mirror the BTE behavior shown in Figure 3b, peaking between 2500 and 2750 rpm for most blends. While low‐concentration blends (B5 to B20) exhibit η_Ex_ values nearly identical to B0, a more discernible reduction in exergetic quality is generally noted as the RSOME fraction increases toward B50 and B100.

**FIGURE 11:**
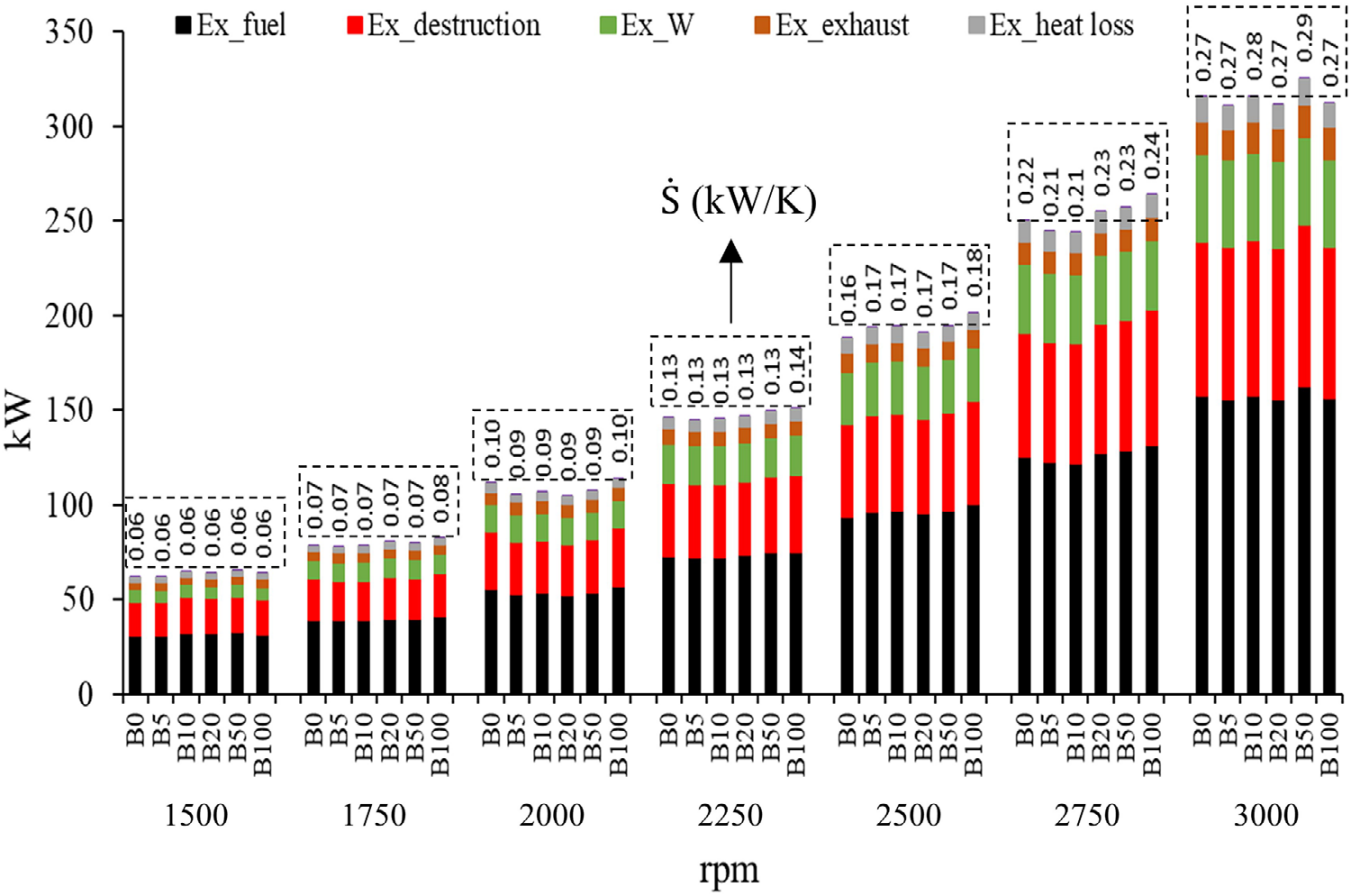
Exergy distribution components (fuel exergy, useful work, exhaust exergy, heat loss exergy, and destruction) for all fuel types, together with the entropy generation rate, $\dot{S}$.

To interpret these differences, the exergy balance is decomposed into its principal components in Figure 11. Accordingly, Figure 11 details the exergy partition, including fuel exergy, useful work, exhaust exergy, heat loss exergy, and exergy destruction for each fuel case. The entropy generation rate ($\dot{S}$), annotated above the columns, shows a clear speed‐dependent increase for all fuels. Specifically, $\dot{S}$ rises from approximately 0.06 kW/K at 1500 rpm to nearly 0.29 kW/K at 3000 rpm, representing more than a fourfold increase in entropy generation. This trend reflects the intensified turbulence, higher mass flow rates, and increased energy throughput at elevated speeds, which collectively expand the shares of exhaust exergy and heat loss exergy.

The impact of the fuel type is predominantly visible at mid‐to‐high speeds. Notably, as the RSOME content in the blend increases, a generally progressive rise in $\dot{S}$ and exergy destruction is observed. This trend is driven by a competition between chemical and physical factors. While RSOME‐rich blends exhibit an advanced combustion onset (lower MBF10)‐primarily due to the high cetane number, which reduces the ignition delay‐this early start is countered by physical constraints such as higher viscosity. The superior ignition quality promotes a more rapid onset of the chemical exergy release, yet the slightly prolonged combustion duration at high speeds increases the time‐dependent entropy generation. However, at the maximum speed of 3000 rpm, a slight deviation from this trend occurs where B50 exhibits the highest entropy generation rate ($\dot{S}$ = 0.29 kW/K), surpassing B100 ($\dot{S}$ = 0.27 kW/K). This suggests that at extreme speeds, the intensified in‐cylinder turbulence and higher energy throughput may interact differently with the fuel's oxygen content and atomization characteristics, allowing B100 to stabilize its irreversibility levels more effectively than B50. Overall, these findings provide a mechanistic explanation for how RSOME blending modifies the pathway of thermodynamic losses without compromising stable brake thermal efficiency, while highlighting that the severity of these losses is sensitive to the specific engine speed regime.

## 4 Conclusion

In this study, Rosehip (*Rosa Canina* L.) seed oil methyl ester (RSOME) was produced and characterized as a novel biodiesel feedstock, revealing a high oxygen content of 11.46% and a superior cetane number of 52.36. Following fuel characterization, the engine performance, combustion characteristics, pollutant emissions, and exergy destruction of a CI engine fueled with diesel (B0), RSOME‐diesel blends (B5, B10, B20, and B50), and neat RSOME (B100) were comprehensively investigated. The key findings derived from the experimental results and thermodynamic analyses, which demonstrate high ignition quality in accordance with international standards, are summarized as follows:

– **Fuel Quality and Efficiency**: The superior cetane number (52.36) and inherent oxygen content (11.46%) of RSOME enhanced the ignition quality and oxidation potential of the test fuels. Based on these advantages, the B5 blend emerged as the most efficient fuel across nearly all engine speeds, improving the BSFC by 1.2% to 5.5% and the exergetic efficiency by 1.6% to 5.9% relative to B0. Notably, these gains were consistently supported by a 1.3% to 8.5% reduction in exergy destruction and comparable entropy generation rates, further demonstrating that B5 maintains superior thermodynamic stability without introducing additional irreversibilities.
– **Combustion Dynamics**: The impact of the B5 blend on combustion performance and phasing was found to be highly speed‐dependent, revealing a transition from physical constraints to chemical advantages relative to diesel. At lower speeds (1500–1750 rpm), B5 exhibited a slight reduction in P_max_ (up to 2.6%) and IMEP (0.8%); although the combustion onset (MBF10) was advanced, higher viscosity slightly hindered atomization under low‐turbulence conditions. Conversely, at the maximum speed of 3000 rpm, the oxygen‐enhanced reactivity of B5 became dominant; the combustion duration (MBF90‐10) was substantially shortened, and the overall combustion phasing was advanced. This shift effectively overcame high‐speed ignition delays, leading to a 6% increase in P_max_ and a 5.4% improvement in IMEP, which directly correlates with the peak exergetic performance observed for this specific blend.
– **Emission Characteristics**: The gaseous emissions of the B5 blend exhibited a strong sensitivity to engine speed, transitioning from a low‐speed disparity to superior performance at high speeds. While B5 produced 32% higher NO_x_ and 18% higher CO than diesel at 1500 rpm, these trends reversed dramatically as engine speed increased. At medium‐to‐high speeds (above 2500 rpm), the B5 blend achieved significant reductions, reaching a 6.8% decrease in NO_x_ and a substantial 65% reduction in CO emissions at 3000 rpm relative to B0. This suggests that at higher turbulence and shorter residence times, the 11.46% fuel‐borne oxygen of RSOME effectively promotes more complete oxidation of carbon, while its latent heat of vaporization and improved combustion phasing mitigate the oxygen‐driven NO_x_ formation.

## Conflicts of Interest

The authors declare no conflicts of interest.

## Data Availability

The data that support the findings of this study are available from the corresponding author upon reasonable request.
